# Sweat Facilitated Amino Acid Losses in Male Athletes during Exercise at 32-34°C

**DOI:** 10.1371/journal.pone.0167844

**Published:** 2016-12-09

**Authors:** R. Hugh Dunstan, Diane L. Sparkes, Benjamin J. Dascombe, Margaret M. Macdonald, Craig A. Evans, Christopher J. Stevens, Marcus J. Crompton, Johan Gottfries, Jesse Franks, Grace Murphy, Ryan Wood, Timothy K. Roberts

**Affiliations:** 1 University of Newcastle, Callaghan, NSW, Australia; 2 Latrobe University, Melbourne, VIC, Australia; 3 School of Health and Human Sciences, Southern Cross University, Coffs Harbour, NSW, Australia; 4 Department of Chemistry and Molecular Biology, University of Gothenburg, Gothenburg, Sweden; Universitat de les Illes Balears, SPAIN

## Abstract

Sweat contains amino acids and electrolytes derived from plasma and athletes can lose 1-2L of sweat per hour during exercise. Sweat may also contain contributions of amino acids as well as urea, sodium and potassium from the natural moisturizing factors (NMF) produced in the stratum corneum. In preliminary experiments, one participant was tested on three separate occasions to compare sweat composition with surface water washings from the same area of skin to assess contributions from NMF. Two participants performed a 40 minute self-paced cycle session with sweat collected from cleansed skin at regular intervals to assess the contributions to the sweat load from NMF over the period of exercise. The main study investigated sweat amino acid composition collected from nineteen male athletes following standardised endurance exercise regimes at 32–34°C and 20–30% RH. Plasma was also collected from ten of the athletes to compare sweat and plasma composition of amino acids. The amino acid profiles of the skin washings were similar to the sweat, suggesting that the NMF could contribute certain amino acids into sweat. Since the sweat collected from athletes contained some amino acid contributions from the skin, this fluid was subsequently referred to as “faux” sweat. Samples taken over 40 minutes of exercise showed that these contributions diminished over time and were minimal at 35 minutes. In the main study, the faux sweat samples collected from the athletes with minimal NMF contributions, were characterised by relatively high levels of serine, histidine, ornithine, glycine and alanine compared with the corresponding levels measured in the plasma. Aspartic acid was detected in faux sweat but not in the plasma. Glutamine and proline were lower in the faux sweat than plasma in all the athletes. Three phenotypic groups of athletes were defined based on faux sweat volumes and composition profiles of amino acids with varying relative abundances of histidine, serine, glycine and ornithine. It was concluded that for some individuals, faux sweat resulting from exercise at 32–34°C and 20–30% RH posed a potentially significant source of amino acid loss.

## Introduction

Various studies have revealed the highly complex nature of the metabolite composition of sweat [1–5]. Amino acids were identified as constituents as early as 1910 [4] and in 1934 it was hypothesised that sweating could result in significant losses of amino acids, although under resting conditions and in cooler climates, the loss of constituents relative to blood-plasma would be relatively low [5]. There is a potential for excessive exercise under warmer conditions to lead to a net negative nitrogen balance with detrimental impacts on muscle recovery and performance [6, 7]. Although it is known that amino acid sweat composition [8] and sweating rates [9] vary greatly among individuals despite similarities in diet, levels of fitness and age [10], relatively little research has been done to determine the impacts of sweat facilitated losses of amino acids (SFLAA).

It has been proposed that a primary source of amino acids in sweat is from blood plasma. However, the amino acid profiles of human sweat and plasma have been shown to differ significantly. Serine, histidine, alanine and ornithine are generally found in high levels relative to plasma whilst relatively low levels of sweat glutamine have been reported [1, 3, 11]. Mark and Harding [12] analyzed the free amino acid content of sweat collected from the surface of the axillae of healthy adults at rest and concluded that the amino acid content of the stratum corneum directly affected the amino acid composition of eccrine sweat with the hydrolysis of the protein fillagrin being a significant determinant. It has recently been suggested that sweat or water could rapidly leach some of the electrolytes from the stratum corneum of the skin [13] while free amino acids have also been measured in samples derived from water washings of human skin that were attributed to the stratum corneum and skin surface film [14]. Natural moisturising factor (NMF) is produced in the stratum corneum, representing 10% of the dry weight of this skin layer, and contains a select group of amino acids and their derivatives [15] such as urocanic acid which is derived from histidine and pyrrolidone carboxylic acid which is derived from glutamine [16]. These amino acids act as humectants maintaining moisture within the stratum corneum. It is proposed that the amino acid content of sweat collected from the skin’s surface would be primarily derived from the eccrine glands with contributions from the leachate of NMF from the stratum corneum. The sweat found on the surface of the skin has previously been termed “faux” sweat [13] on the basis of the proposal that electrolytes would be derived from both the sweat glands and the stratum corneum. If amino acids were leached from the stratum corneum, it would be expected that continued sweating would be associated with reductions in sweat amino acid concentrations over the period of exercise as the NMF leachate would become depleted. If the time of sweat collection was not incorporated into the experimental design, then contributions of the NMF could explain some of the high variance in sweat composition reported previously [1, 3, 11].

In order to understand net sweat-facilitated losses of amino acids during exercise, it was therefore important to undertake a preliminary investigation to assess the potential contributions of amino acids from the NMF to the composition of “faux” sweat. The first objective of the current study was thus to determine whether the skin’s surface could contribute to sweat amino acid loading, and if so, whether these loadings would become more dilute with extended sweating or rinsing of the skin. The contributions of amino acids from NMF were then taken into account for the design of the major study to compare the compositions of amino acids in sweat and plasma from well-trained male endurance athletes and assess the potential sweat-facilitated losses of amino acids. The number of athletes assessed also allowed multivariate investigations of the amino acid composition profiles to assess whether subgroups of athletes could be delineated based upon their sweat characteristics of overall amino acid losses and sweat rates during exercise in relatively hot conditions.

## Materials and Methods

### Preliminary assessments for contributions of amino acids from the skin surface

To assess potential contributions from the stratum corneum, sweat and water washings of skin were collected on three separate occasions at weekly intervals from one male. Sweat was collected three times from the back at 10 minute intervals over the course of a standardised 30 minute exercise routine and combined for comparison with samples collected from skin water washings. The exercise was undertaken in the early evening at 28–32°C, the participant then showered and slept overnight in temperatures ranging from 18–24°C. Twelve hours post-exercise, a wash sample was collected by spraying deionised water onto the skin of the back sufficient to generate droplets for immediate collection of at least 1mL in a sterile specimen container. The participant then showered and towel-dried thoroughly before immediately collecting a second sample by spraying and collecting the droplets from the skin. This approach was designed to indicate whether the stratum corneum could contribute amino acids to water on the skin surface as would be expected if leaching had occurred.

Sweat samples from two athletes were then collected during a steady-state self-paced cycling session lasting 40 minutes. Samples were collected from the forearm at 15 minutes, the skin washed, and then subsequent samples were taken from the same area of skin cleansed at 7–9 minute intervals. This approach was designed to evaluate whether changes in faux sweat concentration occurred over the exercise period to reflect diminishing contributions of amino acids from the stratum corneum as reserves become depleted via the leaching process. Some leachate may have been removed by the washing step. This activity was approved by the University of Newcastle Human Research Ethics Committee (approval number: H-2015-0534) and the participants provided written informed consent prior to inclusion in the study. The same two participants had completed a separate study within 6–8 weeks of performing the current sweat evaluation, and average fasting plasma concentrations were determined over a 6 week period. These data were used as a base reference for comparison with the sweat concentrations in the present study. The project from which the plasma baseline data was sourced was separately approved by the University of Newcastle Human Research Ethics Committee (approval number: H-2015-0401) and participants provided written informed consent prior to inclusion in the study.

### Comparison of sweat and plasma amino acids in well trained endurance athletes

#### Participants, Exercise and Sample Collection

A primary study group was recruited comprising eleven well-trained male endurance athletes (age: 29 ± 9 y, height: 179 ± 7 cm, body mass: 73 ± 10 kg, Σ7 skinfolds: 58 ± 24 mm) who had completed at least ten 5 km competitive runs in the previous two years. Potential participants were excluded if they reported any medical conditions (cardiovascular, musculoskeletal or metabolic) that would have increased their risk of experiencing an adverse event during the exercise. Participants performed three simulated 5 km self-paced time trials after a ten minute warm up, separated by seven days, on a non-motorised treadmill, with various cooling interventions as detailed below in an environmental chamber to provide a constant environment at 32–34°C and 20–30% RH. As part of a larger study [17], participants randomly completed the repeat 5 km self-paced time trials where they either underwent pre-cooling by ingestion of 7.5 g/kg of ice slurry (-1°C, Gatorade, PepsiCo, New York, USA) in the 30 min prior to the run; mid-cooling by a menthol mouth rinse (swilling 25 mL of an 0.01% concentration of an L-menthol solution at 22°C, Mentha Arvensis, New Directions, Sydney, Australia) in the mouth for 5 s prior to expectoration into a bucket, or no intervention. There was no significant effect on sweat composition between these cooling strategies. Multiple plasma and sweat samples from each participant were thus collected and averaged to provide single representative samples for individuals in the current study. This project was approved by the University of Newcastle Human Research Ethics Committee (approval number: H-2012-0311) and all participants provided written informed consent prior to inclusion in the study.

An additional study group comprising eight male triathletes was recruited to provide sweat samples to facilitate extended subgroup evaluations of sweat composition characteristics. These athletes, recruited from the local community, were aged from 25 to 35 years and had completed an Olympic distance triathlon within the preceding 12 months (age: 29.6 ± 3.4 y, body mass: 77.8 ± 11.1 kg, VO_2_max: 62.1 ± 4.9 mL/kg/min, Olympic distance triathlon time in last 12 months: 2:10:12 ± 0:9:12 h:min:s, mean ± SD). Potential participants were excluded if they reported any medical conditions as described above. Participants performed two simulated Olympic distance triathlon trials separated by seven days. The triathlon comprised three standardised legs including a swim (1500 m) in a 50 m indoor pool, a cycle (1 hour) on a cycle ergometer (Lode Excalibur Sport, Groningen, Netherlands) and a 10 km self-paced time trial on a motorised treadmill (Powerjog JM100, Expert Fitness UK, Mid Glamorgan, Wales). The cycle and run legs were performed within an environmental chamber to provide a constant environment at 32–34°C and 20–30% RH. Each participant was required to begin the exercise trial hydrated and was weighed just prior to initiating exercise. During the cycle component, participants ingested either 10 g/kg BM of ice slurry (< 1°C) or room temperature (32–34°C) sports drink (Gatorade, PepsiCo, New York, USA). The methods for the triathlon trials and provision of the sports drink has previously been presented for this cohort [17]. There was no effect on sweat composition between these cooling strategies. The project was approved by the University of Newcastle Human Research Ethics Committee (approval number: H-2011-0024) and all participants provided written informed consent prior to inclusion in the study.

Plasma samples were taken at the pre- and post-exercise sampling times for the primary study group. The results of multiple plasma samples from each participant at repeat sessions were averaged to provide a single representative value from each individual in the study. Sweat samples were collected during both trials by direct collection into a sterile 70 mL specimen jar (Sarstedt, Germany). In the primary study group, each of the eleven athletes provided sweat samples at all three exercise sessions. Sweat was collected from the back immediately after the treadmill run into a sterile specimen container. The samples were stored at 4°C and analysed within 48 hours. In the secondary cohort, five of the eight athletes provided sweat samples on two occasions; on one occasion under conditions of provision of cold slurry and on a second occasion under provision of ambient temperature fluids. Three of the athletes provided only one sweat sample under provision of either cold slurry or ambient temperature fluids. Results from multiple sweat samples from each participant were averaged to provide a single representative value for each individual in the study. Following the exercise trial, the subjects were dried by towel and weighed to determine total fluid loss during the exercise regime. The total sweat volume was calculated as the total body mass lost during the exercise session corrected for fluid and food intake. Sweat samples were analyzed for amino acid composition using the EZ:Faast™ (Phenomenex® Inc.) derivatisation kit for analyses of amino acids by gas chromatography/flame ionisation detection (GC/FID) as previously described by Evans et al., [18].

The samples taken throughout the preliminary investigations to assess potential contributions from the skin surface and the primary athlete study groups to study sweat-facilitated losses of amino acids have been summarized in Table 1.

**Table 1:** Summary of biospecimen collection conditions from the various investigations.

Assessment	Participants	Sweat sample collection site	Sample collection conditions
Assessment of skin surface and sweat amino acids	Male, n = 1 *Sampling tested on 3 separate occasions*	Back	Sweat samples collected 3 times at 10 min intervals during 30 min of exercise. Skin water washings were taken after (1) 12 hrs post-evening exercise after showering and sleeping or (2) immediately after showering following sleep
Sweat amino acids changes during exercise versus baseline plasma amino acids	Male recreational athletes, n = 2	Forearm	After 15 min of a 40 min steady-state self-paced cycling, a sweat sample was collected, the skin washed and a total of 3 samples taken at approx. 8 min intervals. Comparisons were made between sweat and mean plasma amino acid levels taken over a preceding 6 week period
Sweat versus plasma amino acid levels after exercise	Male endurance athletes, n = 11	Back	Sweat samples were taken after three 5 km self-paced runs on a non-motorised treadmill. Plasma samples taken pre- and post-exercise
Sweat amino acid levels after exercise	Male endurance athletes, n = 8	Back	Sweat samples taken after two simulated triathlons

### Statistical Analysis

The different cooling treatments had no effect on amino acid composition of plasma or sweat samples as assessed by ANOVA. Replicate sweat and pre- or post-exercise plasma samples for each athlete were thus averaged to include one representative value for each athlete. Pre- and post-exercise plasma amino acid by one-way ANOVA and levels of statistical significance (alpha) were set at < 0.05. In addition, plasma and sweat compositions between the groups defined on the basis of total amino acid concentrations in the sweat were also assessed via ANOVA (Tukey’s honestly significant difference test for unequal sample sizes). The sweat excretion clusters were analysed using principal component analysis (PCA) on log-transformed concentration data, and correlation analyses were performed using Dell Statistica (data analysis software system), version 13, software.dell.com, Dell Inc. (2015).

## Results

### Assessments for contributions of amino acids from the skin surface

**Fig 1:**
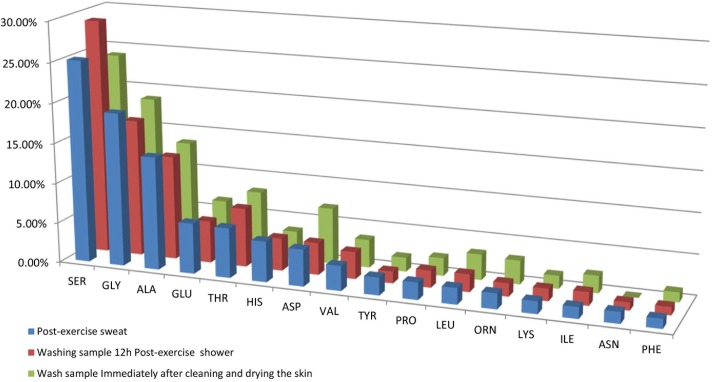
Comparison of the relative (percentage) abundances of amino acids in (a) post-exercise sweat, (b) a water-washing taken after 12 hours rest following a post-exercise shower and (c) a water-washing taken immediately after showering and drying. Values are averages from three separate sampling events from one male participant.

A preliminary study was undertaken to investigate potential contributions to amino acid loading in sweat from the skin surface. The levels of amino acids were measured in sweat collected from the back of one male participant following an evening exercise session on three separate occasions, yielding an average total amino acid concentration of 4.5 ± 1.2 mM. This was then compared with the composition of a water-washing sample for the skin surface taken 12 hours after the post-exercise shower which had a total amino acid content of 1.2 mM, equivalent to 27% of the earlier post-exercise sweat sample. A subsequent skin washing sample collected immediately after showering and drying was found to contain a total amino acid content of 0.18mM, equivalent to 4% of the post-exercise sweat sample. In Fig 1, the percentage relative abundances for these three types of samples indicated that the amino acid composition profile of the fluid from freshly washed skin surfaces mirrored the composition profile of post-exercise sweat.

**Fig 2:**
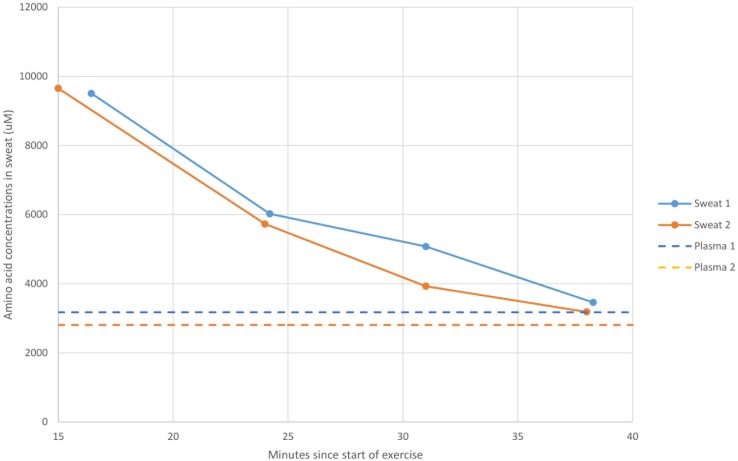
Total amino acid levels (μM) in sweat measured for two athletes completing a 40 minute self-paced cycle under controlled conditions compared against fasted plasma levels previously recorded for each of the athletes over a 6 week period.

A second set of preliminary experiments were undertaken to investigate whether the loading of amino acids from the skin surface would be exhausted by leaching over a period of time. Two athletes undertook self-paced 40 minute cycling sessions where sweat was collected after 15 minutes from the forearm. The skin surface was then washed, and subsequent samples were taken followed by cleansing of the skin at regular 7–9 minute intervals. The evaluation of total amino acids in the sweat was then plotted against time with a comparison of their corresponding fasted plasma amino acid levels in Fig 2. The concentrations of amino acids in the sweat diminished with time reaching levels equivalent to those observed in the plasma after 35 minutes of exercise. It was concluded that the fall in amino acid concentrations in the sweat represented the diminishing contributions from skin to the sweat composition over the period of exercise.

### Comparison of sweat and plasma amino acids in well trained endurance athletes

The primary study goals were to compare amino acid composition of sweat and plasma in endurance athletes during controlled exercise events and assess the levels of sweat-facilitated losses of amino acids. For these athletes, the sweat was either collected at the end of the running session for the 5 km self-paced time trials or at the end of the simulated triathlons. This approach would have minimalized the contributions from the natural moisturizer factors that were initially leached from the skin contributing to the faux sweat, as shown in Fig 2.

Twenty-six amino acids were detected and quantified in the faux sweat collected from the primary athlete group (n = 11) and the results summarised in Table 2 for comparison with corresponding plasma amino acids taken at the same pre- and post-exercise time points. Aspartic acid and hydroxylysine were present in the sweat but were both absent in the pre- and post-exercise plasma samples. The average total concentration of amino acids in faux sweat was more than three-fold higher than those observed in the blood plasma (Table 2). A total of 13 amino acids were present in faux sweat at concentrations significantly (*P* < 0.05) higher than those recorded in the post-exercise plasma: α-amino-adipic acid, asparagine, aspartate, glutamic acid, glycine, histidine, hydroxylysine, isoleucine, leucine, lysine, ornithine, phenylalanine and serine. Four amino acids were present in the faux sweat in significantly lower concentrations compared with the post-exercise plasma: α-amino-butyric acid, glutamine, cystine and proline. In comparison to pre-exercise plasma levels the post-exercise plasma amino acids showed a statistically significant increase in alanine and significant decreases in asparagine, lysine, ornithine, serine, and threonine.

**Table 2:** Comparison of sweat amino acid concentrations with pre- and post-exercise plasma amino acid levels measured in the primary group of male athletes.

Amino acid	Plasma Pre-exercise amino acid μM (mean ± SE) (n = 10)	Plasma Post-exercise amino acid μM (mean ± SE) (n = 10)	Faux sweat Post -exercise amino acid μM (mean ± SE) (n = 11)
α-amino-adipic acid	1.1 ± 1	3 ± 1	74 ± 23 ^b^
α-amino-butyric acid	15 ± 2	12 ± 2	4 ± 3 ^c^
Alanine	375 ± 23	499 ± 23^a^	630 ± 123
Asparagine	39 ± 1	32 ± 2 ^a^	62 ± 10 ^b^
Aspartic acid	0	0	174 ±28 ^b^
β-amino-isobutyric acid	1.4 ± 1	1.6 ± 1	12 ± 7
Cystathionine	6 ±2	3 ± 2	15 ±12
Cystine^e2^	1 ± 1	15 ± 2	3 ± 2 ^c^
Glutamine	430 ± 23	380 ± 19	73 ± 31 ^c^
Glutamic acid	31 ± 3	39 ± 3	200 ± 32 ^b^
Glycine	194 ± 8	193 ± 84	910 ± 169^b^
Histidine^e^	52 ± 3	48 ± 2	1,400 ± 519 ^b^
Hydroxylysine	0	0	67 ± 26 ^b^
Hydroxyproline	4 ± 1	2 ± 1	3 ± 2
Isoleucine^e^	60 ± 3	61 ± 2	158 ± 34 ^b^
Leucine^e^	119 ± 5.6	120 ± 4	295 ± 59 ^b^
Lysine^e^	165 ± 8	142 ± 6 ^a^	637 ± 211 ^b^
Methionine^e^	18 ± 1	19 ± 1	24 ± 10
Ornithine	43± 1	34 ± 2 ^a^	977 ± 335 ^b^
Phenylalanine^e^	45 ± 1	49 ± 2	157 ± 37 ^b^
Proline	230 ± 15	207 ± 9	88 ± 10 ^c^
Serine	77 ± 5	49 ± 4 ^a^	1,240 ± 199 ^b^
Threonine^e^	116 ± 5	89 ± 5 ^a^	147 ± 26
Tryptophan^e^	38 ± 3	31 ± 2	104 ± 34
Tyrosine^e2^	4.7 ± 3	1 ± 1	16 ± 7
Valine^e^	270 ± 14	258 ± 12	250 ±45
**Total**	**2,350 ± 162**	**2,290 ± 151**	**7,790 ± 1,850**

^a^Amino acid levels in the post-exercise plasma were significantly different to pre-exercise plasma levels

^b^ Amino acid levels in the sweat were significantly higher compared with the post-exercise plasma levels

^c^ Amino acid levels in the sweat were significantly lower compared with the post-exercise plasma levels

^e^ Essential amino acids

^e2^Tyrosine can be synthesised from phenylalanine and cysteine (within cystine) can be synthesised from methionine and serine.

Comparison of the amino acid composition of faux sweat was determined for the secondary athlete group (n = 8) and compared with the primary group data (n = 11) which revealed that there were no significant differences in the total levels of amino acids in sweat between the two groups. Only three amino acids, together representing 5% of the composition of sweat, were statistically different between the primary and secondary study groups. These three amino acids were leucine measured in the primary athlete group (295 ± 158 μM vs secondary group 106 ± 28 μM); α-amino-adipic acid (74.5 ± 23 μM vs 6.5 ± 0.6 μM); and tyrosine (15.6 ± 7.4 μM vs 127 ± 32 μM) (*P* < 0.05). The participants’ data were thus combined to form a larger dataset (n = 19) and the data were appraised for obvious differences in sweating characteristics such as amino acid concentrations, sweat volume and total amino acids lost via the sweating process.

**Fig 3:**
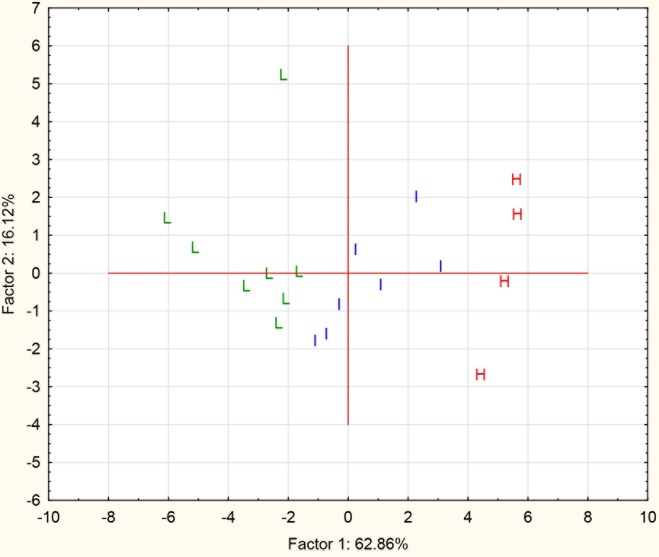
The scatterplot of the principle component analysis (PCA) scores (factor 1 vs factor 2) of the amino acid composition profiles in sweat from the combined study cohort (n = 19). The scores for each of the participants have been coded for membership of one of the three clusters: L = low, I = intermediate, H = high.

When the individuals were ranked on the basis of their total amino acid concentrations in faux sweat, it was possible to place the athletes into three distinct clusters or subgroups characterised by their sweat facilitated loss of amino acids (SFLAA): 1) a “Low” cluster was defined as possessing a total amino acid concentration in sweat of <4.0 mM (n = 8) with a mean ± SD of 2.4 ± 0.7 mM; 2) an “Intermediate” cluster was defined as 4.0 to 10.0 mM (n = 7) with a mean of 5.9 ± 1.7 mM; and 3) a “High” cluster was defined as >10.0 mM^.^(n = 4) with a mean of 15.2 ± 3.3 mM. The ability to categorise the participants in this manner provided an explanation of the high variances observed in the whole combined study group. Multivariate analyses were then applied to the dataset to determine whether qualitative differences in the amino acid composition of sweat would differentiate group membership. In Fig 3, factors generated by principle component analysis (PCA) fully resolved the members of the three clusters based on the patterns of amino acid composition in the sweat from each individual.

The sweat characteristics and amino acid compositions of faux sweat from each of the three clusters have been summarised in Table 3. The “Low” SFLAA group displayed the highest estimated sweat volume per hour at 2.3 L/h and the lowest total amino acid concentration in sweat at 2.4 mM with an estimated quantity of amino acids lost per hour via sweat at 5.1 mmoles. In contrast, the “High” SFLAA group had the lowest estimated sweat volume per hour at 1.5 L/h and the highest total amino acid concentration at 15.2 mM with an estimated quantity of amino acids lost via sweat per hour at 22.8 mmoles (Table 3). The “Intermediate” SFLAA group’s sweat volume per hour of 1.8 L/h and total sweat amino acid concentration of 5.9 mM fell between the “High” and “Low” group values with an estimated quantity of 10.6 mmoles lost via sweat per hour.

**Table 3:** Comparison of amino acid concentrations in sweat from the “Low”, “Intermediate” and “High” SFLAA clusters compared with the post-exercise composition of plasma.

	Post-exercise plasma amino acid concentrations (P) (μM ± SE)	Sweat amino acid concentrations per SFLAA cluster (μM ± SE)			Sweat—Plasma concentrations (μM)		
Amino acid	Primary group (n = 10)	Low (L) Total amino acids <4 000 (n = 8)	Intermediate (I) Total amino acids 4,000 to 10,000 (n = 7)	High (H) Total amino acids >10,000 (n = 4)	L-P	I-P	H-P
Serine	49 ± 4	582^a^ ± 135	1,160^b^ ± 111	2,410^d^ ± 359	533	1,111	2,361
Glycine	193 ± 84	349^a^ ± 36	682^b^ ± 78	1,590^c^^,^^d^ ± 146	156	489	1,397
Alanine	499 ± 23	235^a^ ± 28	457 ^b^ ± 40	1,170^c^^,^^d^ ± 73	-264	-42	671
Histidine^e^	48 ± 2	212^a^ ± 54	1,010 ^b^ ± 379	3,300^d^ ± 986	164	962	3,252
Ornithine	34 ± 2	151^a^ ± 33	1,340 ^b^ ± 352	2,150^d^ ± 530	117	1,306	2,116
Aspartate	0	119^a^ ±18	251 ± 33	322 ±130	119	251	322
Lysine^e^	142 ± 6	104 ± 31	414 ^b^ ± 152	1,340^d^ ± 352	-38	272	1,198
				*Sub-totals*	*787*	*4*,*349*	*11*,*317*
Threonine^e^	89 ± 5	117 ± 27	212 ± 33	250 ± 108	28	123	161
Valine^e^	258 ± 12	99^a^ ± 8	190 ^b^ ±27	445^c^^,^^d^ ±24	-159	-68	187
Leucine^e^	120 ± 4	87 ± 20	212 ^b^ ± 51	477^d^ ± 74	-33	92	357
Glutamic acid	39 ± 3	68 ± 23	196 ^b^ ± 24	378^c^^,^^d^ ± 67	29	157	339
Proline	207 ± 9	59^a^ ± 8	93 ± 9	156^d^ ± 38	-148	-114	-51
Glutamine	380 ± 19	58^a^ ± 16	42 ± 16	163 ± 71	-322	-338	-217
Phenylalanine^e^	49 ± 2	48 ± 10	122 ^b^ ± 29	277^d^ ± 41	-1	73	228
Isoleucine^e^	61 ± 2	47 ± 8	114 ^b^ ± 21	288^d^ ± 35	-14	53	227
Tyrosine^e2^	1 ± 1	34^a^ ± 13	72 ± 36	103 ± 59	33	71	102
Asparagine	32 ± 2	20 ± 6	60 ^b^ ± 9	114^c^^,^^d^ ± 19	-12	28	82
Tryptophan^e^	31 ± 2	11^a^ ± 6	52 ^b^ ± 17	215^d^ ± 57	-20	21	184
α-aminoadipic acid	3 ± 1	9 ± 5	27 ± 10	153^d^ ± 36	6	24	150
Hydroxylysine	0	2 ± 1	23 ^b^ ± 10	171^d^ ± 36	2	23	171
Hydroxyproline	2 ± 1	0	14 ± 6	3 ± 2	-2	12	1
				*Sub-totals*	*-613*	*157*	*1*,*921*
Total Amino Acid Concentrations in Post-exercise Plasma and Sweat (mM)	2.2 ± 0.15	2.4 ± 0.26	5.9^b^ ± 0.63	15.2^c^^,^^d^ ± 1.6			
				*Sub-totals*	*0*.*2*	*3*.*9*	*13*.*0*
Resting Total Amino Acid Concentrations in Plasma (mM)		2.45 ± 0.25	2.39 ± 0.09	2.24 ± 0.11			
Estimated total sweat volume per hour exercise period, L/hour, (n = 11)	Primary Group	2.3 ± 0.30 (n = 4)	1.8^f^ ± 0.33 (n = 3)	1.5^g^ ± 0.1 (n = 3)			
Estimated total amino acids lost/hour exercise period, mmoles, (n = 11)	Primary Group	5.1	10.6	22.8			

^a^ The concentrations of amino acids in sweat for the “Low” cluster were significantly different compared with corresponding levels in the plasma (*P* < 0.05). The sweat parameters were assessed by Tukey’s HSD for unequal N where

^b^ Intermediate > Low

^c^ High >Intermediate

^d^ High > Low (P<0.05)

^f^ Intermediate<Low

^g^ High<Low.

^e^ Essential amino acids

^e2^Tyrosine can be synthesised from phenylalanine and cysteine (within cystine) can be synthesised from methionine and serine.

The first seven amino acids segregated in Table 3 represented those with the highest losses in faux sweat for any of the three SFLAA clusters. In the “Low” cluster, the total amino acid concentration of faux sweat was equivalent to the plasma level. However, subtraction of the plasma levels from the sweat levels (Table 2, L-P) revealed that most of the seven high loss amino acids were present at much higher concentrations, with serine ten times higher than in the plasma. Aspartic acid was not detected in plasma but was present as the sixth most abundant amino acid in the faux sweat from the “Low” cluster and was observed at higher concentrations for the remaining groups. In contrast, eight of the remaining 14 amino acids measured in the faux sweat from the “Low” cluster were lower than the plasma levels and included five essential amino acids.

The difference in concentrations of the seven sweat amino acids present in excess compared with plasma was 787μM. In contrast, the difference between faux sweat and plasma levels of the remaining amino acids (including essential amino acids) represented a shortfall by 613μM. In the “Intermediate” cluster, the excess of the seven amino acids was 4,349μM and the losses of the remainder amino acids were still kept to a minimum at a total of 157μM in sweat with only three amino acids less than plasma levels (Table 2, I-P). The “High” cluster had an even greater excess of the most abundant seven amino acids in the sweat, with excesses observed in all other amino acids except for glutamine and proline which were present in lower concentrations in the sweat compared with plasma for all groups (Table 2, H-P).

**Fig 4:**
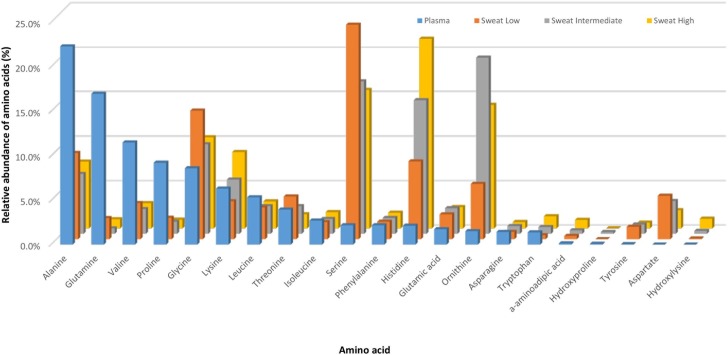
The relative abundances of amino acids in blood plasma ranked from the most abundant to least abundant components compared with those observed in sweat from the “Low”, “Intermediate” and “High” SFLAA clusters.

The differences in amino acid profiles between the three clusters were apparent in terms of concentrations as well as relative abundances of each of the components in the profiles as shown in Fig 4. The order of amino acids have been ranked from the highest to lowest relative abundances of amino acids present in the plasma to contrast the differences between plasma and faux sweat concentrations. The “Low” SFLAA cluster was characterised by having in order of greatest abundance: serine, glycine, alanine and histidine which accounted for 57% of the amino acid composition of the faux sweat; the “Intermediate” cluster had ornithine, serine, histidine and glycine as the major components comprising 71%; the “High” cluster had histidine, serine, ornithine and glycine comprising 62% of the sweat amino acid composition. The total amino acids in resting plasma levels were highest in the “Low” SFLAA cluster and lowest in the “High” SFLAA cluster, and although the differences were not significant, a strong negative correlation was observed between the resting total plasma concentrations and the total sweat concentrations (*r* = -0.99, ie the higher the amino acid concentration in sweat, the lower the resting concentration of amino acids in plasma).

## Discussion

The faux sweat collected in the present study was characterised by high levels of serine, histidine, ornithine, glycine, alanine, and lysine which have previously been reported at concentrations several times higher than that of plasma [1, 3, 11], while aspartic acid was detected in sweat but not in the plasma. The high values of these amino acids were consistent with leaching of amino acids from the skin surface initially contributing to the amino acid load in sweat, as previously suggested for electrolytes, to form “faux” sweat [13]. Natural moisturizing factor is produced in the stratum corneum where its constituent amino acids are thought to be derived almost entirely from the protein filaggrin, with serine, histidine, arginine/ornithine, glycine, glutamine/glutamic acid, and aspartic acid/alanine as the major components [19]. Burke and Lee (14) also demonstrated that serine, glycine, alanine and histidine were the most abundant amino acids in skin water washing taken from the torso. However, it was shown in the current study that total amino acid levels in sweat diminished during 35 minutes of exercise (Fig 2), falling to levels equivalent to those measured in plasma. The preliminary results of the present study therefore supported the hypothesis that sweat can leach amino acids from the stratum corneum which may account for the relatively high levels of amino acids measured in faux sweat in the early stages of sweating. It was proposed that sweat taken after approximately 30–35 minutes of exercise would contain minimal contributions from NMF. The secondary group of triathletes underwent 2 hours and 15 minutes of exercise, including a swim stage, and there were no significant differences between the levels of the major seven amino acid sweat components in this and the primary set of athletes, further supporting the notion that the contributions from NMF were minimal after 35 minutes of exercise. Further studies should, however, be made to determine whether rates of NMF production and replenishment may vary between individuals and also between different locations on the body.

In an endeavour to explain the high variance for the sweat data, the athletes were partitioned into three clusters based on ranges of amino acid concentrations in faux sweat which resulted in groups with differential sweat volumes and composition profiles of amino acids. As would be expected, those with higher sweat volumes had lower sweat amino acid concentrations compared with those with lower sweat volumes who had higher concentrations in sweat. The average total levels of the pre- and post-exercise amino acid concentrations in the plasma were not significantly different which suggested that the athletes maintained hydration and amino acid homeostasis in the circulating plasma. The total concentration of amino acids in faux sweat from the “Low” SFLAA cluster had an average level equivalent to the plasma concentration. The concentrations of amino acids in faux sweat from the “Intermediate” and “High” SFLAA cluster athletes were much higher than the plasma levels, which were not accounted for by their lower sweat volumes.

Since glutamine and proline were always lower in the faux sweat compared with the plasma levels, it was concluded that that filtering or reabsorption of these amino acids occurred to reduce their losses via sweating as previously proposed by Liappis et al. [20]. In the “Low” cluster, it appeared that valine and tryptophan were also effectively conserved by similar mechanisms. To date there have been no reported mechanisms for selective concentration of amino acids into sweat from plasma in the secretory cells of the eccrine sweat glands. It was thus concluded that the loading of amino acids in faux sweat must have come from the skin and that the differences between the 3 groups of athletes would be characterized by the levels of NMF present at the skin surface as well as the rates of replenishment of NMF. This would need to be tested in the future by carefully assessing concentrations in skin washings from members of the different clusters.

Each cluster had a characteristic composition of the predominant amino acids in the faux sweat: serine, ornithine or histidine. The faux sweat amino acid data were subjected to PCA which objectively separated individuals based on their amino acid profiles. When individual cases were coded for cluster membership, it was clear that the amino acid patterns in the faux sweat were characteristic for each cluster with a complete resolution of the three groups. Each cluster had a different composition profile regarding the seven major amino acid components (Fig 4) providing evidence for the presence of differential contributions from the skin for the members of each cluster. Individual variations in the levels of NMF production or replenishment rates combined with variations in the mix of key amino acids in NMF could provide a mechanism to explain the differences observed in the faux sweat amino acid profiles for the clusters. Although current projections of potential sweat losses per hour did not include initial contributions from NMF, differences in sweat composition from different skin locations, nor errors induced by evaporative fluid losses, they did provide a basis for comparison and estimating initial nutrient losses via faux sweat. The “High” SFLAA cluster was estimated to lose 22 mmoles of amino acids per hour of exercise compared with 5.1 mmoles lost by the “Low” cluster. The level of concentration of amino acids in the lower sweat volume from the “High” cluster represents a very intense concentration of amino acids via the sweating and leaching processes.

The relatively elevated levels of sweat-facilitated losses of amino acid losses observed in the “High” cluster compared with the other two clusters suggested that many nonessential amino acids may become conditionally essential [21, 22] during prolonged exercise when the body may not be able to meet demands via synthesis. The classification of athletes as members of the “High” SFLAA cluster may therefore identify those that require a higher level of amino acid support to sustain exercise and training. Based on World Health Organization recommendations, it was calculated that the loss of histidine in faux sweat during the exercise regime for the “High” SFLAA cluster members may have represented up to 40% of their recommended daily allowance of 10 mg^/^kg/day [23]. In contrast, the “Low” SFLAA cluster could be proposed as a phenotype best suited for exercise under warm conditions at 32–34°C where there is a high fluid loss for efficient cooling but reduced losses of amino acids. The extent of amino acid losses also indicated a necessity to ensure a balanced intake of appropriate amino acids to address losses in sweat and to maintain nitrogen balance.

Future research will aim to develop nutritional supplements formulated to compensate for the major losses resulting from eccrine sweat and the amino acids leached from skin surfaces in the early stages of exercise. In a previous study, provision of a complex amino acid supplement to males from the general public resulted in reduced levels of fatigue [24] which provided some evidence that fatigue may be associated with a net deficit in the nitrogen balance. In another recent study investigating sweat losses in Standardbred horses during race training, provision of amino acids targeted to replace specific sweat-facilitated losses resulted in elevated resting plasma levels which could assist the support of high intensity training [25]. The athletes in the present study had resting plasma total levels of amino acids which were lower than general population literature values [26].

To meet the demands for amino acids during exercise, the body adopts a catabolic response allowing it to access non-myofibrillar proteins in muscle [27, 28]. Catabolism of myofibrillar proteins can occur under conditions of prolonged and strenuous exercise to satisfy the body’s demand for amino acids, potentially resulting in muscle damage. This muscle tissue damage, which can in turn limit muscle performance leading to peripheral fatigue [29], has been associated with high-intensity exercise and over-training [28]. If plasma volume is assumed to be 3 L, then the total quantity of amino acids in circulation can be calculated as being between 6.7–7.35 mmoles. In this context, the impact of losing 5.1–22.8 mmoles via eccrine sweating of amino acids over an hour may place substantial demands on catabolic processes to release amino acids into circulation in order to maintain homeostasis and to support metabolism and recovery during exercise. Although the quantity of amino acids lost through sweat may represent a relatively small proportion of the average daily intake of protein, these losses occur at a time of elevated proteolysis in the muscles to support exercise [28]. Future amino acid support strategies would include the development of supplements containing free amino acids for direct absorption (bypassing digestion), that could be taken during and immediately after exercise to counter catabolic demands on muscle proteins.

## Conclusion

The initial composition of sweat included contributions of key amino acids from the skin’s NMF which were similar in profile to the major components measured in sweat. The contribution from the skin surface diminished after 35 minutes of self-paced exercise. The amino acid composition of faux sweat collected from the major study participants post-exercise, with minimal contributions from NMF, were significantly different to the amino acid composition of plasma. The amino acids serine, histidine, ornithine, glycine, alanine, aspartic acid and lysine were identified as major components in sweat, present in substantially higher levels in faux sweat compared with the plasma levels. It was proposed that the high levels of these amino acids in faux sweat were derived from NMF in the skin, and that the differentiation between the three clusters involved differential capacities to produce and replenish NMF during exercise. Glutamine and proline concentrations in sweat were always lower compared with plasma suggesting mechanisms of filtration or re-absorption. Each cluster of athletes had characteristic sweat volumes as well as amino acid profile characteristics which could be differentiated by PCA. It was proposed that those individuals making up the “High” SFLAA cluster would be placed at a greater risk of amino acid depletion during exercise in warmer conditions than those belonging to the remaining two clusters. Phenotypic variation between the three amino acid loss clusters may involve variable capacities for fluid retention, production of NMF and mechanisms for filtration and/or reabsorption of key amino acids.
